# Application of Intensity-Based Coherent Optical Time Domain Reflectometry to Bridge Monitoring

**DOI:** 10.3390/s22093434

**Published:** 2022-04-30

**Authors:** Xin Lu, Sebastian Chruscicki, Marcus Schukar, Sven Münzenberger, Katerina Krebber

**Affiliations:** Bundesanstalt für Materialforschung und -prüfung, Unter den Eichen 87, 12205 Berlin, Germany; sebastian.chruscicki@bam.de (S.C.); marcus.schukar@bam.de (M.S.); sven.muenzenberger@bam.de (S.M.); katerina.krebber@bam.de (K.K.)

**Keywords:** structural health monitoring, distributed fiber sensing, distributed acoustic sensing, destructive testing

## Abstract

Although distributed fiber sensing techniques have been widely used in structural health monitoring, the measurement results of bridge monitoring, particularly under destructive testing, have rarely been reported. To the best of our knowledge, this paper is the first report of distributed vibration measurement results, which we obtained during a three-day destructive test on an abolished bridge. A coherent optical time domain reflectometry (COTDR) was used to acquire the vibration information while the bridge was being sawed. The obtained signal was analyzed in time and frequency domain. Some characteristics of the sawing-induced vibration were retrieved by the short-time Fourier transform; the vibration exhibited several high frequency components within the measured range up to 20 kHz and all the components appeared in the same time slot. Some unexpected signals were also detected. Thorough analysis showed that they are quite different from the sawing-induced vibration and are believed to originate from internal damage to the bridge (probably the occurrence of cracks).

## 1. Introduction

Various types of optical fiber sensors have been widely applied to structural health monitoring thanks to the intrinsic advantages of the optical fiber, such as small size, chemical inertness, immunity to electromagnetic interference, and so on [1]. Recently, the distributed optical fiber sensor has attracted more attention, as it can perform seamless measurement over a certain range, unlike the discrete sensor, which is unable to acquire the information between two sensing points. This advantage is of particular interest for structural health monitoring, as small cracks may happen at any position in the structure. However, the sensitivity of the distributed sensor is usually not as good as the discrete sensor [2].

Distributed fiber sensors (DFSs) rely on various backscattering mechanisms in the fiber and perform the spatially resolved measurement over the whole fiber length. The Raman-based DFS exploits the optical power change of the anti-Stokes component of the spontaneous Raman scattering to retrieve the temperature information. The DFS based on Brillouin scattering usually uses the Brillouin frequency shift to measure temperature and strain with a typical sensitivity of 1 MHz/K and 0.05 MHz/με, respectively, and at an operating wavelength of 1550 nm [3]. Since strain is a very important parameter to evaluate the structural condition, the Brillouin-based DFS is an effective tool for structural health monitoring. The Brillouin-based DFS has been applied to monitor the condition of bridges [4] and tunnels [5]. It also has many applications in geophysics monitoring of ground displacements [6], landslides [7,8], and so on. Although the measurement time is very long, as the sensing system usually needs frequency scanning to acquire the Brillouin frequency spectrum, the Brillouin-based DFS can also realize dynamic measurement by exploiting the linear slope of the Brillouin spectrum [9]. However, the low sensitivity of the sensor fails to meet the requirement of many applications.

A distributed acoustic sensor (DAS), also known as coherent optical time domain reflectometry (COTDR), retrieves the environmental information based on the interference result of the Rayleigh backscattered light [10]. It fulfils the requirement of both high sensitivity and short measurement time. It demonstrates high sensitivities of ~1.3 GHz/K for temperature and ~150 MHz/με for strain [11], which are three orders of magnitude higher than the Brillouin-based sensor. However, the response time of the sensing system is only limited by the fiber length; for example, the sensor bandwidth is about 50 kHz for a sensing distance of 1 km. The upper limitation can be surpassed by hardware modification or an advanced sampling algorithm [12,13]. In addition, the current COTDR system has the ability to detect dynamic strain in the level of pε/√Hz. As a result, the COTDR system is an excellent candidate for dynamic measurement over long distances. For example, the COTDR system has gained success in seismic event detection in the ocean and glaciated terrain [14,15], and it has been applied to structural health monitoring, particularly in the oil and gas industry monitoring boreholes, production processes, and pipelines.

The COTDR system is meant to find many applications in structural health monitoring. Particularly, it can be used to determine the vibration frequency of a bridge and provide a warning if the bridge vibrates close to its natural frequency. Such a sensor has the potential to measure the acoustic emission, allowing the cracks in the bridge to be detected [16]. The sensor can also measure the load, and even the traffic over the bridge. However, the application of the COTDR to bridge monitoring is at its infant stage, and only a few on-site tests have been reported to the best of our knowledge. Cheng et al. reported a dynamic load result measured by a phase-based DAS system at a bridge in the Netherlands [17]. We have used a wavelength-scanning COTDR system for dynamic strain measurement at a 24.4 m-long bridge model [18].

In this paper, we report the measurement results using an intensity-based COTDR system during a destructive testing of a bridge in Germany. More information about this bridge can be found in [19]. This is the first application of such a simple DAS system to bridge monitoring to the best of our knowledge. The testing was performed before the demolition of the bridge, so aggressive testing methods were possible; therefore, the presented results are unique. The inner wall of the bridge was sawed in order to introduce cracks or fractures. Our system measured the vibration signal during the sawing process, and the obtained result was analyzed thoroughly. Spectrum analysis revealed that some vibration behaved differently from the sawing-induced events. The results reported here show that the COTDR system can locate and record the vibration events in a bridge, thus it has the potential to warn of damages. Due to different causes, the detected signal can exhibit various frequency characteristics. As a result, a COTDR system can provide meaningful signals that can help civil engineers identify various occurrences inside the bridge, making it possible to realize structural health monitoring for the bridge.

## 2. Working Principle of an Intensity-Based COTDR System

Rayleigh scattering originates from the density variation of the medium that is formed during the fiber drawing process, and it accounts for most of the fiber loss at 1550 nm. A COTDR system sends narrow and highly coherent optical pulses into the sensing fiber and collects the interference result of the Rayleigh backscattered light. Due to the stochastic distribution of the fiber density, the interference process is totally random and changes with the fiber position, thus the obtained COTDR trace exhibits a noise-like shape along the fiber [20]. Although the obtained signal is totally random, the signal remains the same and repeatable under the identical condition for the same fiber. Therefore, the DAS trace can be considered as the “fingerprint” of the fiber. External perturbations such as temperature and strain variations, however, modify the local interference condition so that the obtained signal is changed. The first intensity-based COTDR system was demonstrated in 1993, and it could detect the intrusion based on the signal change [21]. The system obtains the Rayleigh backscattered light directly from the sensing fiber and obtains the COTDR trace continuously during the measurement time. External perturbations can be spatially determined based on the location of the trace change, and the frequency information can also be retrieved. As a result, the DAS system is an excellent tool for distributed vibration monitoring.

The intensity-based COTDR system fails to quantify the measurand because the signal change has not even a monotonic relationship with the environmental variations [22]. Hence, the system has been improved in the last decade to quantify strain. However, the classical system is still an attractive solution to many applications due to its simple configuration and data processing. As a result, an intensity-based COTDR system was built to acquire the bridge vibration information during the destructive testing.

## 3. Materials and Measurement Method

**Figure 1:**
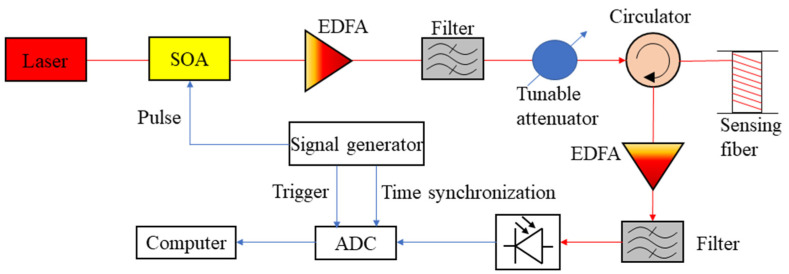
Configuration of the distributed acoustic sensor used for bridge destructive testing. The red line represents optical path and the blue line denotes the electrical connection.

The configuration of the sensing system used for bridge monitoring is depicted in Figure 1. A semiconductor laser (RIO ORION, Santa Clara, CA, USA) with a linewidth of 2.9 kHz was used as the light source. A signal generator provided rectangular pulses to a semiconductor optical amplifier (SOA) so that the continuous wave from the laser was modulated by the electrical pulse and converted into optical pulses with a high extinction ratio. Then, the generated pulses were boosted by an erbium-doped fiber amplifier (EDFA) and a narrowband filter was employed to filter out the amplified spontaneous emission (ASE) from the amplifier. The peak power of the pulse had to be adjusted by a tunable attenuator to avoid non-linear effects in the sensing fiber. The pulse entered the sensing fiber through a circulator and the Rayleigh backscattered light was directed to the receiving module by the same circulator. As the backscattered light is very weak, another EDFA was employed as a pre-amplifier. The amplified light passed through another narrow filter and arrived at a photodetector.

The pulse width was set as 10 ns during the measurement, corresponding to 1 m spatial resolution. The bandwidth of the photodetector was 125 MHz, which could guarantee the spatial resolution [23,24]. The pulse repetition rate was 40 kHz, so the frequency response of the system was up to 20 kHz. An A/D convertor (ADC) synchronized with the signal generator was used to digitalize the output of the detector at a rate of 500 MS/s. The digitalized data was processed by a computer to retrieve the vibration information.

**Figure 2:**
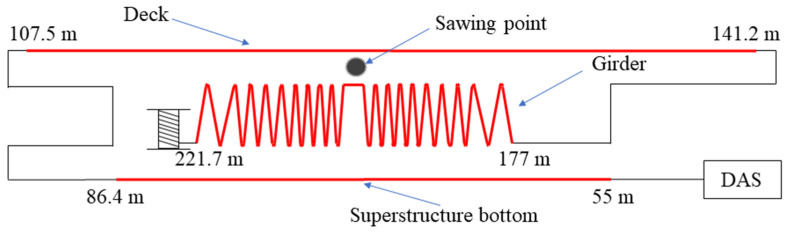
Optical fiber deployment in the bridge. The red line indicates the sensing sections.

The superstructure of the concrete bridge was ~37 m wide and over 150 m long. There were several box girders in the main beam with a height of ~1.5 m. Detailed information is provided in [19]. A standard single mode fiber with a 3 mm-diameter jacket was used for the measurement, and its length was in total ~245 m. The optical fiber was attached to the bridge as shown in Figure 2 for sensing. The whole fiber section from 55 m to 86.4 m was glued to the bottom of the superstructure. Another section from 107.5 m to 141.2 m was buried in a slot on the deck. In addition, a ~44.7 m-long fiber between 177 m and 221.7 m was totally glued in a zigzag shape on the inner wall of the girder. A rear handle saw was used to cut a rebar in the inner wall of the girder. The whole measurement was performed without any traffic.

## 4. Results and Discussion

For each measurement, the sawing process lasted several minutes; however, the sensing system continued to acquire data over tens of minutes. Hence, many results were obtained during the three-day test, and only a few exemplary results are presented here.

### 4.1. Sawing-Induced Vibration

**Figure 3:**
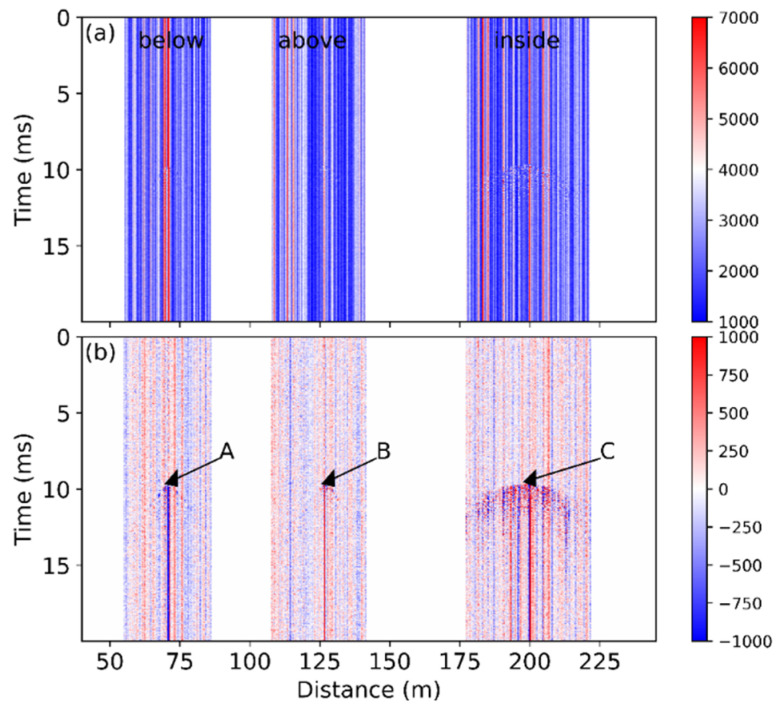
(**a**) Raw data and (**b**) data change compared with the first obtained trace during the sawing process. Points A, B, and C indicate the starting positions of the vibration.

**Figure 4:**
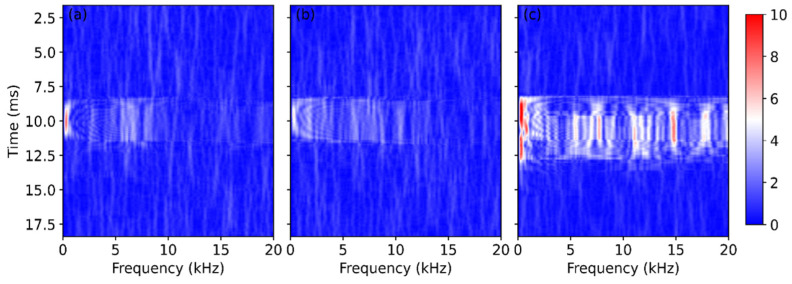
Temporal evolution of the frequency spectra at the first signal change points (**a**) at the bottom of the bridge; (**b**) above the deck; (**c**) inside the girder.

The raw data presented in Figure 3a were also analyzed in the frequency domain by the short-time Fourier transform (STFT). The STFT is an effective tool for feature extraction of the obtained signal and has been applied to identify the threats to gas pipelines [25]. At each position, the frequency spectrum of a window of 128 temporal points was obtained, and this operation was repeated while the window slid in time domain. As a result, a group of spectra was obtained at each fiber position over the measurement time, and the STFT result *S*(*t*, *z*, *f*) is actually a 3D matrix in time, distance, and frequency domain. Figure 4 shows the STFT results of the A, B, and C points, where the first signal variation was observed at each fiber section.

The figure exhibits significant signals at ~10 ms due to the sawing process. For the fibers installed below and above the bridge, most of the signal was below 10 kHz, as shown in Figure 4a,b, respectively. In comparison, the fiber attached inside the girder measured a signal over the whole frequency range up to 20 kHz, and the received signal was much stronger, as shown in Figure 4c.

Although the measured frequency spectra vary from case to case, the last fiber section always experiences the most intense vibration, as demonstrated by Figure 3 and Figure 4. This can be explained in two aspects. Firstly, the fiber in the girder is the closest to the sawing position, so it experiences the strongest and probably longer perturbation. The mechanical vibration becomes less intense during its propagation across the bridge, resulting in a weak influence on the fiber above and below the bridge. However, the fiber is glued on the vertical wall in the girder, whereas the other sensing sections are placed horizontally, as shown in Figure 2. The vibration may travel vertically, in parallel with the fiber in the girder, thus most of the fiber experiences large perturbation because the fiber is most sensitive to the axial strain. However, the working principle of the COTDR system is much less sensitive to the radial strain [26]; the signal obtained from the other fiber section therefore varies mildly.

**Figure 5:**
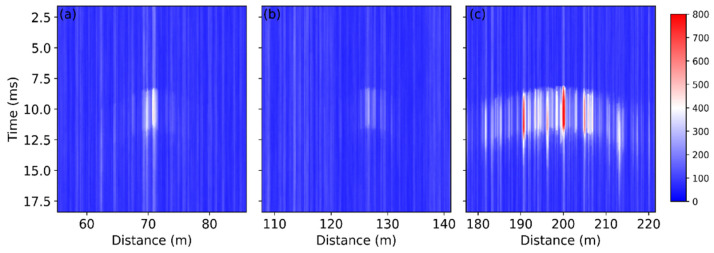
Temporal evolution of the frequency signal summed from 0 to 20 kHz (**a**) at the bottom of the bridge; (**b**) above the deck; (**c**) inside the girder.

The frequency analysis also helps identify the vibration events. The STFT results within the frequency range between 0 Hz and 20 kHz are summed up at each position and time point. The obtained results for each fiber section are plotted in Figure 5, respectively. The ratio between the peak value (~7000) and the standard deviation of the data (~1278) obtained inside the girder as shown in Figure 3a is about 5.5. On the contrary, the ratio is over 10 for Figure 5c. As a result, the vibration shown in Figure 5 is visually easier to identify than Figure 3, and the edges are more obvious. The frequency result might therefore be more helpful to analyze the vibration behavior, such as the wave propagation speed.

**Figure 6:**
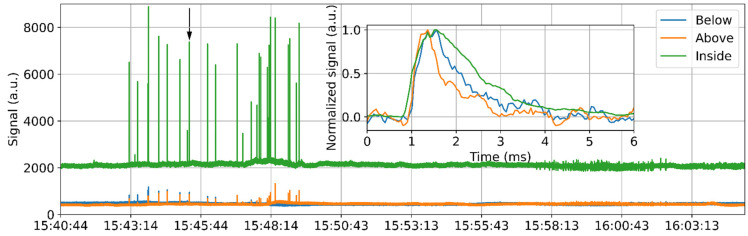
Temporal evolution of the summation result over the whole sensing section. Inset: zoom-in of one peak as indicated by the arrow; the DC level is removed and the peak is normalized to its maximum.

Another usage of the frequency result is to detect the effective sawing during the whole measurement. In this case, the STFT result over each sensing section and the whole frequency range is summed, so the obtained data changes only with time. The summation results for all three sensing sections are plotted in Figure 6 over a 20 min measurement time. Since the sensing fiber inside the girder is longer than other sections, the signal obtained from this part has a larger value, so the corresponding DC level is much higher than that of the other fiber sections. The sawing process started at about 15:43 and lasted about 6 min. Many peaks are shown during this time period in Figure 6, which are supposed to be caused by the interaction between the saw teeth and the rebar during the sawing process. Ideally, the peaks should have appeared at a fixed time interval as the saw blade rotated. However, the saw was held by hand and therefore moved during the sawing process, which caused the quasi-random occurrence of the peaks, as shown in Figure 6. The peak obtained inside the girder exhibits a very high value, as this fiber section is longer than the others and closest to the cutting point, as explained above.

The inset in Figure 6 shows the temporal evolution of one peak as indicated by the arrow; the DC is removed, and the peak is normalized to its maximum value for a better visualization. The green curve, representing the signal from the girder, starts to increase a bit earlier than other curves, as this fiber section is the closest to the sawing point. In addition, the corresponding signal decreases more gradually due to the long fiber section inside the girder. The occurrence time difference of different fiber sections may be very useful to locate the vibration source in practice. It is not likely that the actual vibration always occurs in proximity to the sensing fiber. By analyzing the time delay of the peaks detected by different fiber sections, the origin of the vibration can be located with a high accuracy. A similar method has been applied to determine the breakdown discharge position in a gas-insulated switchgear [27].

### 4.2. Unexpected Vibration

**Figure 7:**
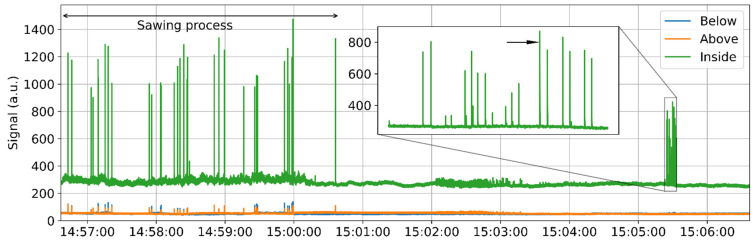
Temporal evolution of the summation result over the whole sensing section. The destructive testing process lasted ~4 min; then, an unexpected signal is detected, as shown in the inset.

The STFT result as shown in Figure 6 seems to be an effective tool to detect the occurrence of the sawing and was used to analyze all the data obtained during the three-day testing. Figure 7 shows another measurement over 10 min, and the sawing-induced signal peaks can also be clearly observed during the destructive testing process, which lasted ~4 min. Interestingly, the sensing fiber is vibrated ~1 min after the sawing, as the obtained signal varies slightly between 15:02 and 15:03, as shown in Figure 7. The fiber section is then perturbated significantly, as the inset clearly shows many peaks within a period of 10 s, which have lower amplitudes than the sawing-induced signal. This is totally unexpected, as the bridge was free from any external perturbation at that time. Hence, this acoustic event could only have been triggered by some internal factor. A further and detailed analysis is necessary to determine the cause of this vibration.

**Figure 8:**
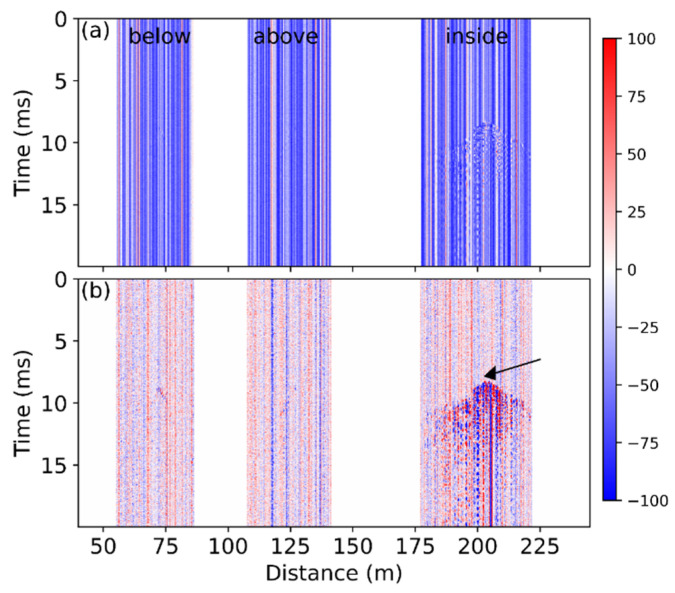
(**a**) Raw data of the unexpected signal and (**b**) data change compared with the first obtained trace. Arrow denotes the starting position of the vibration.

One of the unexpected acoustic events, as signified by the arrow in the inset, was thoroughly investigated. The raw data and the data change are plotted in Figure 8, and the vibration signal can only be observed clearly for the fiber section inside the girder. The arrow in Figure 8b indicates the starting point of the signal; it occurs at ~203.5 m, about 3.7 m away from the detected sawing position. However, the actual location of the vibration is closer to the sawing position as indicated by the zigzag shape; the location of this event is different from the sawing case. In addition, the spread of the vibration is different from the sawing-induced vibration. The boundary of the perturbated region as shown in Figure 8b is steeper than Figure 3b shows, indicating the propagation speed of this signal was much smaller than the sawing-induced signal. One possible explanation is the occurrence position of the vibration. The sawing-induced vibration mainly propagated along the reinforced steel; the speed of the sound in such a material is comparatively large. The unexpected vibration may occur inside the concrete, where the corresponding sound speed is smaller.

**Figure 9:**
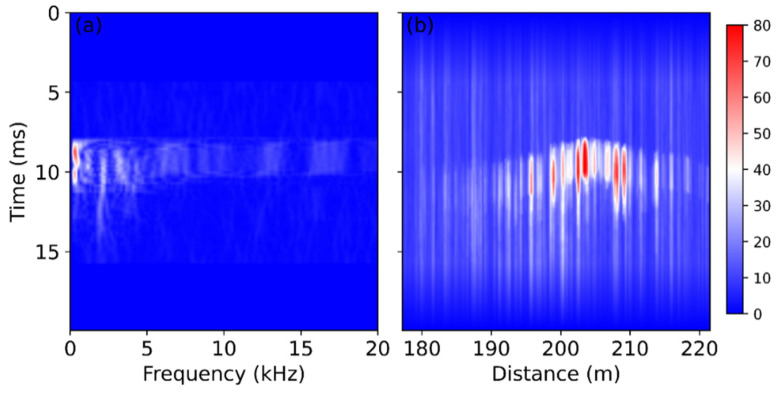
Temporal evolution of (**a**) the frequency spectra at 203.5 m and (**b**) the frequency result summed from 0 to 20 kHz over the whole sensing section.

The signal from the fiber section inside the girder was further analyzed in frequency domain. The STFT result of the signal at the starting point (203.5 m) is shown in Figure 9a. Most of the energy associated with this unexpected vibration is at a low frequency, below 1 kHz, unlike the sawing-induced perturbation, which exhibited several high-frequency components according to Figure 4c. The STFT result summed within the frequency range between 0 Hz and 20 kHz is shown in Figure 9b, and it exhibits large amplitudes at several positions. In comparison, the sawing-induced vibration shows a high amplitude only at the starting point, as presented in Figure 5c. The different behaviors may be dependent on the origin of the vibration. A very strong and large event may occur in the girder, so that the high amplitudes have been observed at many positions, as shown in Figure 9b. Further study on this signal might provide a concrete explanation.

## 5. Conclusions

This paper reports the results of an intensity-based COTDR system measured during destructive testing of a bridge. The sawing-induced vibration was thoroughly investigated in time and frequency domains, and a handful of valuable results were obtained which can aid in bridge health analysis. In practice, the sensing fiber will be deployed as a net to cover most of the bridge. Thus, many fiber sections can measure the same vibration at slightly different times. The propagation speed and the location of the vibration can be determined by the position of the fiber section and the difference in the detection time. In addition, the COTDR system acquired unexpected signals which exhibited different characteristics from the sawing-induced signal. The job of the COTDR system is to detect and locate any vibration events, which may be related to damages inside the bridge. This system can also provide meaningful signals in time and frequency domains so that civil engineers can identify the events based on the detected signal, making the structural health monitoring of the bridge possible.

The COTDR system has also been demonstrated as an effective tool for traffic monitoring in our previous publication [28], and the traffic-induced vibration has features that are different from the damage-induced vibration. Thus, the system has the potential to simultaneously monitor traffic and the structural health of a bridge without either task affecting the performance of the other.

## Data Availability

The data presented in this study are available on request from the corresponding author.
